# Co-existence of Autism Spectrum Disorder (ASD) traits in an Irish sample of adults diagnosed with ADHD. Longitudinal outcomes.

**DOI:** 10.1192/j.eurpsy.2025.1191

**Published:** 2025-08-26

**Authors:** D. Adamis, N. Langan, B. Gavin, F. McNicholas

**Affiliations:** 1 Psychiatry, University College Dublin, Dublin; 2 Sligo Mental health Services, Sligo; 3 University of Galway, Galway; 4 University of Limerick, Limerick; 5 University College Dublin, Dublin, Ireland

## Abstract

**Introduction:**

Autism Spectrum Disorder (ASD) and ADHD are both neurodevelopmental disorders which share genetic heritability and often coexist in adults diagnosed with ADHD and vice versa. Despite the overlap between the two disorders there are enough phenomenological differences to indicate that these conditions are sufficiently distinct

**Objectives:**

To estimate the coexistence of ASD traits in an adult sample diagnosed with ADHD; to compare those screening positive for possible ASD to those scoring negative, in terms of functionality, quality life and clinical outcomes; to explore the effects of ADHD medication in three main outcomes (clinical, quality of life, and functionality) in those with only ADHD and in those with a coexistence of ASD and ADHD.

**Methods:**

Prospective longitudinal study of an adult sample diagnosed with ADHD. Data collected on age, gender, medications and on scales: Autism Spectrum Quotient (AQ-10); Adult ADHD Clinical Outcome Scale (ACOS); Adult ADHD Quality of Life Questionnaire (AAQoL); Weiss Functional Impairment Rating Scale (WFIRS).

**Results:**

Sample of 165 participants was recruited. The AQ-10 showed that n=74 (44.8%) of the participants had traits of ASD. Longitudinal analyses demonstrated that people with ADHD and ASD traits have worse clinical outcomes, quality of life, social skills and family functioning compared to those with ADHD only. The effects of ADHD medications (stimulants, atomoxetine) were significant in the three examined outcomes across the time but no significant effects of medications to those with ASD traits was found.

**Conclusions:**

Hight coexistence of ADHD and ASD traits. Perhaps lesser if clinical diagnosis for ASD was performed. Medications for ADHD did not improve those with ADHD and ASD traits. Service implications (for local services): neurodevelopmental clinics is necessity now and not only special for ADHD, where adults with ADHD and ASD can be diagnosed and treated accordingly.

**Disclosure of Interest:**

None Declared

